# Epidemiologic Modeling with FluSurge for Pandemic (H1N1) 2009 Outbreak, Queensland, Australia

**DOI:** 10.3201/eid1709.102012

**Published:** 2011-09

**Authors:** Philip R.A. Baker, Jiandong Sun, James Morris, Amanda Dines

**Affiliations:** Author affiliations: Queensland Health, Brisbane, Queensland, Australia (P.R.A. Baker, J. Sun, A. Dines, J. Morris);; Queensland University of Technology, Brisbane (P.R.A. Baker, J. Sun);; University of Queensland, Brisbane (A. Dines)

**Keywords:** viruses, influenza, human influenza, influenza A virus, pandemic (H1N1) 2009, pandemic, hospitalization, outbreak, preparedness, hospital planning, forecasting, Queensland, Australia, research

## Abstract

At the beginning of the pandemic (H1N1) 2009 outbreak, we estimated the potential surge in demand for hospital-based services in 4 Health Service Districts of Queensland, Australia, using the FluSurge model. Modifications to the model were made on the basis of emergent evidence and results provided to local hospitals to inform resource planning for the forthcoming pandemic. To evaluate the fit of the model, a comparison between the model’s predictions and actual hospitalizations was made. In early 2010, a Web-based survey was undertaken to evaluate the model’s usefulness. Predictions based on modified assumptions arising from the new pandemic gained better fit than results from the default model. The survey identified that the modeling support was helpful and useful to service planning for local hospitals. Our research illustrates an integrated framework involving post hoc comparison and evaluation for implementing epidemiologic modeling in response to a public health emergency.

## Abstract 2

TOC summary: Predictions of the effect of pandemic influenza on hospital resources were helpful for service planning.

Influenza pandemics can result in substantial excess illness and death (*1**,**2*). In the past century, 3 influenza pandemics have occurred, commonly referred to as the 1918 Spanish flu, the 1957 Asian flu, and the 1968 Hong Kong flu. It is estimated that 40–50 million persons died during the 1918 pandemic, which is considered to be one of the most severe disease events in history (*1**,**2*). The following 2 relatively mild pandemics caused approximately 2 million (1957) and 1 million (1968) deaths, respectively (*1**,**2*).

It is widely foreseen that excess illness and deaths in a future pandemic may place serious demands on and even exhaust the available hospital resources in a community. For example, modeling studies consistently predict that current intensive care unit (ICU) services in several industrialized countries could be overwhelmed during a future event of pandemic influenza (*3**–**5*). The prediction of the expected impact of an emerging pandemic would enable appropriate preparation to be made without diversion of excess resources and thus have the potential to reduce pandemic- and nonpandemic-related illness and death.

Since April 2009, a new variant of influenza virus A (H1N1) initially discovered in Mexico and the United States has caused a wave of pandemic influenza. On May 8, 2009, the first case of pandemic (H1N1) 2009 influenza in Australia was confirmed in Queensland (*6*). During the initial “Delay” and “Contain” phases of the Australian Health Management Plan for Pandemic Influenza (*7*), during April 26–June 22, 2009, a total of 593 laboratory-confirmed cases were notified in Queensland. Among the patients these cases represent, 16 hospitalizations and no deaths were reported (*8*). However, the reported number of cases, hospitalizations, and deaths may only represent a small fraction of the true numbers because not all persons who are infected seek medical care and have a specimen collected. Further, not all specimens will have positive results and be reported (*9*).

To assist hospital planners in their preparation for pandemic (H1N1) 2009, at the emerging stage of the pandemic in 2009, regional epidemiologists made predictions of the potential need for general hospital resources and ICU services for 4 Health Service Districts in Queensland, Australia, by using the FluSurge model developed by the Centers for Disease Control and Prevention in the United States (*10**,**11*). A follow-up survey was conducted in early 2010 to evaluate the application of these predictions. The aims of the studies were to describe the modeling work, to explore the fitness of the predictions to the actual hospital data, and to evaluate the application and usefulness of the modeling to hospital planners.

## Methods

### Design

This article describes results from 2 studies. The first study compared the model’s predictions to the actual data for the largest district, Metro North. The modeling techniques used to calculate these predictions are described. The second study was a Web-based cross-sectional survey among the hospital staff who had access to and used these predictions in the response to the pandemic. The aim of this survey was to examine how the projections were applied and whether they were perceived as useful in planning.

Modeling was undertaken during May 29–June 29, 2009, when cases in Australia emerged. The Web-based survey was conducted in May 2010.

### Service Provision Area

Central Regional Services is 1 of 3 regional services of Queensland Health that provide public health services to 4 Health Service Districts, including Metro North (the northern side of Brisbane, population 770,000), Sunshine Coast-Wide Bay (SCWB, population 501,000), Central Queensland (CQ, population 189,000), and Central West (CW, population 12,000). Together these districts account for 38% of Queensland’s population and 32% of Queensland’s area. According to the remoteness structure defined by the Australian Bureau of Statistics (*12*), most of Metro North and SCWB are classified as major city or inner regional (urban), and CQ and CW are mainly outer regional or remote areas.

### Modeling Tool

FluSurge (*11*) predicts the surge in demand for hospital-based services during an influenza pandemic, yielding estimates of the number of hospitalizations (including ICU admissions) and deaths caused by a pandemic in comparison to the existing hospital capacity. Major assumptions of the FluSurge model include that hospital admissions for pandemic influenza pose an extra inconvenience to the current resources and that the admissions are normally distributed over a given time period of the pandemic. Prior to 2009, the model was used to predict the demand for hospital-based services of a future pandemic in the United States (*3**,**10*), England (*4*), Mexico (*13*), and the Netherlands (*14**,**15*). More recently, this tool was also used in the Australian state of Victoria for the pandemic (H1N1) 2009 (*16*).

### Data Sources

District and age-specific population data (estimated resident population 2007 data from Australian Bureau of Statistics, released August 19, 2008; cat. no. 3235.0) and district-specific hospital resource data (for both public and private hospitals) were used as model inputs. Resource data for public hospitals were obtained from the Monthly Activity Collection produced by the Health Statistics Centre and verified by hospital managers. Private hospital data were based upon licensed capacity of operational status by designations, obtained from the Private Health Unit. As Queensland data on ventilators were unavailable, we used the proportion (53.3%) of available ventilators among available ICU beds in New South Wales (Health New South Wales, unpub. data) to estimate the number of available ventilators in Queensland hospitals. To compare modeling predictions with actual data, weekly numbers of hospital admissions because of pandemic (H1N1) 2009 by care type (general bed care, ICU admission and/or ventilation) for each district were extracted from EpiLog, a Web-based application on which hospital-admitted patients who were suspected of having pandemic (H1N1) 2009 were registered.

### Postservice Survey

We conducted a postservice survey using a Web-based survey tool, SurveyMonkey (*17*), seeking information on 1) users of the predictions (including position, role, and district); 2) use of the predictions; 3) perceived usefulness of the predictions; and 4) recommendations and suggestions. Both closed and open-ended questions were used.

Thirty-one hospital planning staff from the 4 districts who were involved in the pandemic response for their district in June 2009 were approached; 16 (52%) responded to the survey. Data analysis was conducted with 15 respondents (48%) as 1 questionnaire was incomplete. Among the respondents, 6 (40%) were from Metro North, 4 (27%) from SCWB, 3 (20%) from CW, and 2 (13%) from CQ. A large percentage of respondents (40%) held nursing positions. Many other respondents were pandemic planning directors and coordinators (13%), or infection control officers (13%). All played a role in the district pandemic (H1N1) 2009 planning and response.

### Data Analysis

Modeling efforts were summarized, and the main inputs and results of an example district (Metro North) were presented in a descriptive manner. The rationale and procedure of modifications to the default FluSurge assumptions were also reported. Modeling predictions and the actual data from the EpiLog were compared visually by plotting.

Responses to survey questions were presented in count and percentages. Content analysis was used to examine the open-ended questions and examples were provided.

### Ethics Approval

Ethical approval was obtained from the Royal Brisbane and Women’s Hospital Human Research Committee for the survey. Participation in the survey was voluntary. Anonymity, confidentially, and privacy of participant responses and any personal details were assured. All participants completed the consent section before responding to the questionnaire.

## Results

### Modeling for Planning

In the initial modeling stage (May 20–29, 2009) predictions regarding the potential hospital load caused by pandemic (H1N1) 2009 in the 4 districts, were made by using FluSurge 2.0 (www.cdc.gov/flu/tools/flusurge). Given that data regarding the current pandemic were unavailable, we used assumptions derived from previous pandemics in the United States, as recommended by the authors of FluSurge (*11*) and other studies (*4*; Health New South Wales, unpub. data). Assumptions included a 25% attack rate and 12-week outbreak duration (Table).

**Table:** Modeling attempts for pandemic (H1N1) 2009, Metro North Health Service District, Queensland, Australia, 2009*

Variable	FluSurge 2.0	Modified model
Parameter and assumption/source		
Population	812,941	812,941
Gross attack rate	25%	15%
Hospitalization rate	Default	0.5%
Duration of hospitalization	12 wk	14 wk
Proportion of patients needing ICU care	20%	10%
Proportion of patients needing ventilation	15%	7.5%
Average length of non-ICU hospitalization	5 d	5 d
Average length of ICU stay	10 d	10 d
Average length of ventilator usage	10 d	10 d
Available hospital resources	Private and public hospitals	Public hospitals only
Main output		
Hospital admissions	2,840 (range 1,104–3,810)	610
Patients needing general beds only	–	549
Patients needing ICU care	–	61
ICU patients needing ventilator care	–	46
Bed demands/availability during peak week		
General	9%	2%
ICU	83%	10%
Ventilator	117%	13%

*ICU, intensive care unit; –, not applicable.

**Figure 1:**
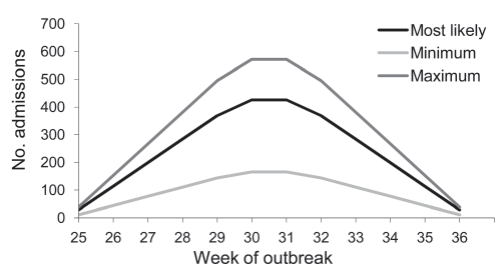
Predicted hospital admissions during an influenza pandemic with 25% attack rate and 12-week duration in Metro North Health Service District, Queensland, Australia, estimated by using FluSurge 2.0.

Using the largest district, Metro North, as an example, we found by initial modeling that a moderate pandemic would result in a total of 2,840 hospital admissions (Table). Peak hospital admission would likely occur at week 6 and week 7 with 426 (range 166–571) admissions per week (Figure 1). During the peak week of the pandemic, 9% of available general beds and 83% of available ICU beds, in both public and private hospitals, would be occupied by patients with pandemic (H1N1) 2009 (Table). The model also predicted that the need for ventilator capacity would exceed the current availability by 17% (Table).

In early June, these results were then presented to those involved in pandemic response and/or hospital planning. However, it was evident through discussion that some major assumptions of the initial model, such as the attack rate, hospitalization rate, and proportions that require ICU or ventilation care, needed revision in line with emerging evidence. For example, although at that time Australia was still in the early stage of responding, data emerging from North America suggested that the new pandemic was much less severe than originally expected. Specifically, it appeared that the present attack rate and incidence of both hospitalization and related death was lower than that used in the model (*18**,**19*; Australian Government Department of Health and Ageing, unpub. data). In addition, historic seasonal influenza data for Queensland suggested that the duration would be longer than 12 weeks (*20*). However, a limitation of the initial model was that the FluSurge only provided 3 options for the attack rate (15%, 25%, and 35%), 3 options for the duration (6, 8, and 12 weeks), and an unadjustable hospitalization risk (*11*).

A modified model with more flexible inputs was then developed to address these limitations. In this modified model, the number of cases related to pandemic (H1N1) 2009 was estimated by total population multiplied by the gross attack rate (same as the original FluSurge model). The number of hospital admissions was then calculated by multiplying the number of cases by the gross hospitalization rate. The modified model also assumed that the total number of admissions would be allocated to each day within the pandemic period according to a symmetric distribution. The number of admissions and bed demands by care type and time were estimated according to the original FluSurge model methods. The main advantage of the modified model was that any value of attack rate (0%–100%), hospitalization rate, and duration could be accepted. However, because a single hospitalization rate was used instead of the default range in the original FluSurge model, the modified model could no longer generate a confidence interval. Nevertheless, the modifications were expedient and addressed the planners’ concerns of unreasonable default assumptions.

On the basis of the modified model, repeated analyses were conducted in June 2009. We hypothesized a 15% attack rate, 0.5% hospitalization rate (among infected persons), and 14-week duration (Table). A series of sensitivity analyses were also made by increasing or decreasing the value of major assumptions within the model. The main results under the base-case assumptions are presented in the Table. At the peak week of hospitalization, the required numbers of 3 types of beds were predicted to account for 2% general, 10% ICU, and 13% ventilator of the current availabilities of public hospital resources (Table). The modified assumptions and results, along with sensitivity analyses were endorsed by Metro North planners and used to inform resource planning.

### Comparisons with Actual Data

We determined that, according to the number of hospital admissions, the 2009 outbreak of pandemic (H1N1) 2009 in Metro North started with week 25. During a 14-week period (weeks 25–38), a total of 308 patients with confirmed or probable pandemic (H1N1) 2009 influenza were hospitalized in Metro North hospitals. The majority (92.3%) were admitted into public hospitals. Seventy-four (21.1%) patients were cared for in an ICU, and 42 (13.6%) were treated with mechanical ventilation.

**Figure 2:**
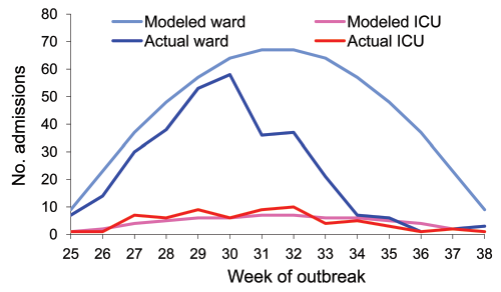
Modeled numbers of total and intensive care unit (ICU) admissions caused by a hypothesized 14-week influenza outbreak in Metro North Health Service District, Queensland, Australia. This model uses assumptions of a 15% attack rate and 0.5% hospitalization rate compared to actual data.

Compared with actual data above, the original FluSurge model (Figure 1; Table) largely overpredicted the effects on hospital-based services. This supported the appropriateness of modifying the assumptions as proposed. The comparison between predictions of the total and ICU admissions based on the modified model and the actual data are shown in Figure 2. This figure shows that the predicted and actual hospitalizations mapped fairly closely for the first 6 weeks of the pandemic, after which there was a rapid drop. Of particular interest to hospital managers was that the actual number of ICU admissions for each week fitted reasonably well to the modeling line (Figure 2).

### Follow-up Survey among Users

Twelve (80%) respondents reported they had used the epidemiologists’ modeling results to inform pandemic response in their districts. Two broad types of use were identified: specific use in bed planning and general use to enhance awareness about the pandemic. Participants from larger districts (Metro North and SCWB) tended to use the results for preparation purposes in bed use, service demand, and staffing. The districts with smaller populations used the models to enhance general awareness of the pandemic.

Four (36%) of 11 respondents believed the predictions had made a change in the pandemic response, 6 (55%) stated no change was induced, and 1 (9%) was not sure. The changes seemed subjective and indirect, derived mainly from better understanding and more confidence among the planners themselves, which in turn would benefit the health system and society. One respondent described how the modeling provided a robust indication of the impact on hospital resources that facilitated changes of work patterns, transfer of patients, and the delay of elective survey. The curve helped identify outbreak progress and when the peak had passed. However, the perceived changes only occurred in the 2 districts with larger populations, most notably Metro North where most of the epidemiologic support was provided. Respondents from the 2 smaller districts did not report any change attributed to the modeling.

All respondents (n = 11) agreed that the predictions were useful to some extent. Four persons considered the predictions to be “quite useful or very useful”; all were from Metro North or SCWB. Participants from the smaller districts (CQ and CW) only considered the modeling to be little to moderately useful.

Similarly, all respondents (n = 14) stated that the communication (e.g., meetings, telephone calls, and emails) between epidemiologists and districts during the pandemic was important to some extent. Nine rated communication as “quite important” or “very important”; 8 were from the 2 larger districts. All respondents (n = 14) agreed that it is helpful to make some predictions before or at the early stage of a pandemic. Most (11/14) believed it was “quite helpful” or “very helpful”; 9 of these respondents were from the larger districts.

Most (13/14) stated that they would recommend predictive modeling be used in future pandemic responses. The modeling assisted hospital planning and enhanced confidence. One respondent identified the role of having trust in the predictions by understanding the assumptions used by the epidemiologists, and that this trust empowered more sophisticated planning and action to manage the excess demand.

## Discussion

We have illustrated a practical framework for epidemiologic modeling in response to a public health emergency, such as a pandemic of infectious disease. The framework could be described as 4 consecutive steps: need identification, modeling and presentation, field use of predictions, and evaluation. This project directly translated existing and emerging knowledge into practice and combined 2 studies to “tell a whole story.”

Although models such as FluSurge are readily available and simple to use, our experience suggests modification is required. When we used modified assumptions on the basis of emergent information on the novel pandemic influenza, more reasonable predictions resulted than when we used default assumptions derived from previous pandemics. Although many modeling studies have been conducted using similar tools (*3**,**4**,**10**,**13**–**15*), none of them have been subsequently tested by using actual data and evaluated in terms of the usefulness in practice. Similar to our initial modeling using the default FluSurge model, most of these studies substantially overestimated the impact of the new pandemic compared to the actual 2009 situation. Lack of communication with users and relying only on historical data may contribute to this problem. The interest of users and their involvement appears to be essential for successful epidemiologic modeling.

The post hoc survey is unique to this study. Results indicate that the modeling and associated support by epidemiologists were well received overall. However, the perceived usefulness of the modeling was more notable for the larger districts than for the smaller districts. We speculate that this occurs because there are larger uncertainties regarding modeling and relatively lower consequences in a small population. Although we did not attempt to measure any direct benefits to the communities, the survey data and ad hoc comments received shows that the modeling and consultation services provided by the epidemiologists were used to manage hospital services confidently.

Although they fitted better, the modified projections seemed to still overestimate the impact, especially the total number of hospitalizations (Figure 2). The main reason is that our assumption of hospitalization rate in general population (15% attack rate × 0.5% hospitalization rate = 75/100,000 persons) is far higher than the actual rate in Metro North (308/770,000 = 40/100,000 persons) and the rate in Australia (23/100,000 persons) (*21*). The pandemic (H1N1) 2009 outbreak turned out to be shorter than observed in seasonal influenza (*20*). One plausible explanation is that Metro North is a metropolitan area and disease transmission there may be more intense than in the whole state, which covers a considerable rural population.

It is unknown whether the awareness of the modeling results might have resulted in changes in admission policy and subsequently affected the number of admissions. In this paper we only presented Metro North data as an example to demonstrate our modeling work. For SCWB district with similar number of residents to the Metro North district, similar results were observed (data not shown). However, for the other 2 small districts, the projections were far more serious than the actual events. This, in accordance with the survey data, indicates that the modeling work for small populations is more difficult and less useful.

There are some limitations to the modeling. First, we assumed a normal distribution of patients over a given period, but the epidemic curve is not symmetric (*22*). The curve usually increases quickly, almost exponentially, and then declines with a long tail after the peak. Second, estimates were made on the basis of the population in these Health Service Districts. The actual number of hospitalizations, however, was obtained from all hospitals within this area rather than all episodes occurring in the whole population. In other words, patient flow was not taken into account in the comparisons. Nevertheless, parallel data obtained from the Queensland Hospital Admitted Patient Data Collection (which records information about episodes of hospital care for all Queensland hospitals) showed that assumptions of patient flows were reasonable and consistent because the net effect was similar. Third, the ventilator capacity was not exactly measured but estimated on the basis of data from another state (*17*), and ICU staff, surge capacity of staff, and absenteeism during a pandemic were not taken into consideration. Furthermore, some major assumptions, such as expected attack rate and hospitalization rate, were somewhat arbitrary. Additionally, many factors that may affect the probability of hospital admission (*23*), such as obesity, pregnancy, and proportion of Indigenous population, were not included in the model. The above limitations would have certain negative effect on the accuracy of the predictions. However, we relied on the best information available at the time. In addition, a series of sensitivity analyses were attached to the base-case estimation. We perceive that the process integrating communication–feedback–presentation is of high value, even higher than the results themselves. The evaluation survey revealed many planners took the opportunity to understand modeling processes and enhance their understanding of the new pandemic.

Another limitation is that only visualization was used to compare projected to actual data, rather than more sophisticated statistical tests. The low response rate (52%) and the small number of respondents (n = 15) may also bias the findings from the survey because nonresponders’ views may differ from responders and thus reduce the representativeness of the sample. Although difficult to assess, the role stated by the participants appears to indicate that key informants from each Health Service District are represented in the survey. The survey is also limited in that several months had passed between when the models had first been applied and when the survey was conducted. Consequently, the survey relied on recall and reflection, both of which can be subject to bias.

Given the above limitations, the net robustness of the model and evaluation are less than ideal, and the predictions and modifications cannot be immediately extrapolated to other areas or outbreaks. Despite these limitations, we firmly believe the principles of modeling and partnerships shown here are valuable for epidemiologists and policy makers in practice and research. It is hoped that the framework illustrated in this study may serve as a general model for epidemiologists who provide epidemiologic services in a public health unit context.
